# In silico design and evaluation of a multiepitope vaccine against *Bordetella pertussis*: structural, immunological, and biological properties

**DOI:** 10.1186/s44342-025-00049-0

**Published:** 2025-07-01

**Authors:** Negar Souod, Hamid Madanchi, Fariborz Bahrami, Saeed Reza Pakzad, Fereshteh Shahcheraghi, Soheila Ajdary

**Affiliations:** 1 Department of Immunology, Pasteur Institute of Iran, Tehran, IR Iran; 2 Department of Medical Biotechnology, Faculty of Medicine, Semnan University of Medical Sciences, Semnan, IR Iran; 3 Nervous System Stem Cells Research Center, Semnan University of Medical Sciences, Semnan, IR Iran; 4 Vaccine Potency and Standardization Section, Food and Drug Control Laboratory (FDCL), Ministry of Health and Medical Education, Tehran, IR Iran; 5 Department of Bacteriology, Pasteur Institute of Iran, Tehran, IR Iran

**Keywords:** *Bordetella pertussis*, Multiepitope vaccine, Mucosal immunity, C-CPE, Immunoinformatics

## Abstract

**Introduction/objectives:**

Despite widespread vaccination, the increasing incidence of pertussis underscores the urgent need for innovative vaccine strategies. This study aims to design and analyze, using in silico methods, a multiepitope protein that incorporates epitopes from the S1 subunit of pertussis toxin and the type 1 immunodominant domain of filamentous hemagglutinin (F1). The goal is to enhance both systemic and mucosal immunity through the incorporation of the C-terminal fragment of *Clostridium perfringens* enterotoxin (C-CPE).

**Methods:**

Using reverse vaccinology, we predicted immunogenic epitopes for lymphocytes derived from the S1 and F1 proteins. The epitopes were assembled into a multiepitope construct named mF1S1-C-CPE, which was then evaluated for its physicochemical, immunological, and biological properties. Immunoinformatics tools were employed to analyze antigenicity, allergenicity, and population coverage. Additionally, molecular docking simulations of peptide‒MHC and mF1S1-C-CPE_TLR2/TLR4 binding were conducted.

**Results:**

Structural analysis indicated that the final multiepitope construct maintained stability and solubility in aqueous environments. Immunoinformatic analysis revealed strong immunogenic properties, effectively eliciting both systemic and mucosal immune responses. Molecular docking demonstrated high-affinity binding patterns between the peptides (both individual or within the mF1S1-C-CPE) and corresponding HLA molecules. Additionally, molecular docking simulations of mF1S1-C-CPE and TLR2/TLR4 indicated strong binding affinity to receptors of innate immunity. The construct was predicted to be stable, soluble, and suitable for expression in *Escherichia coli* (CAI 0.93; GC content 54.9%).

**Conclusion:**

This innovative approach holds promise for enhancing pertussis vaccination strategies by improving mucosal immune responses. Further in vivo studies are essential to validate the efficacy of this multiepitope vaccine candidate.

**Graphical Abstract:**

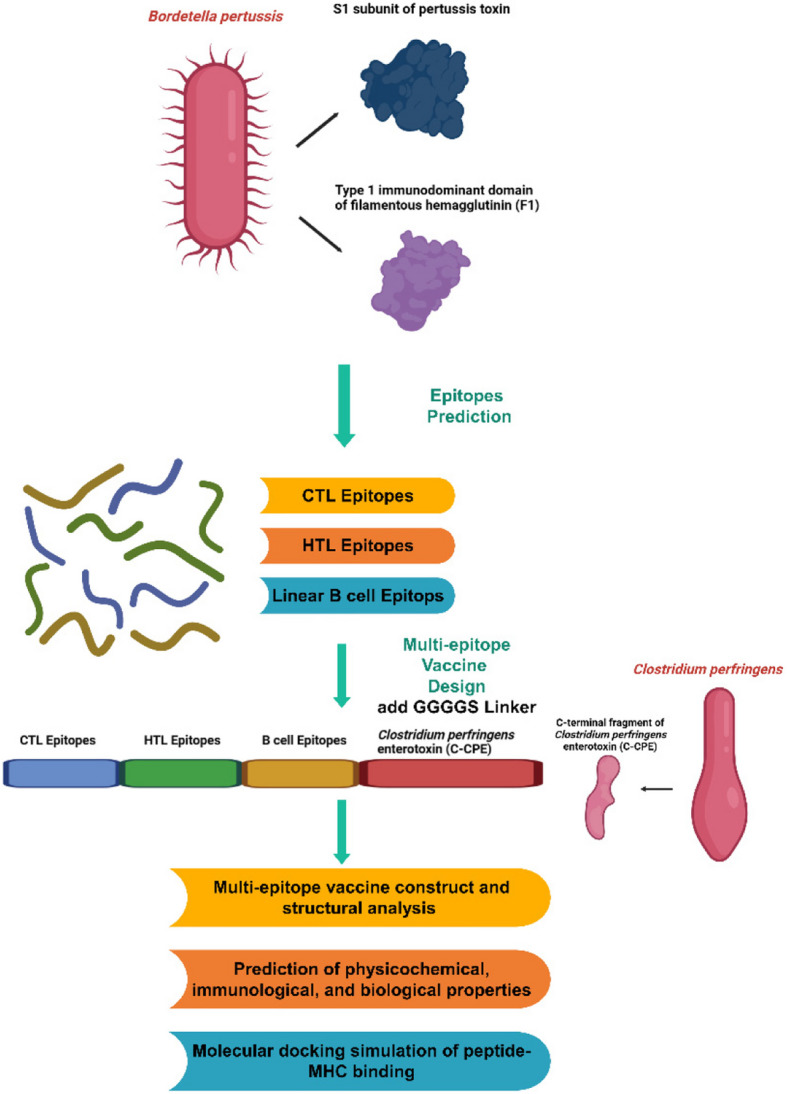

**Supplementary Information:**

The online version contains supplementary material available at 10.1186/s44342-025-00049-0.

## Introduction

Despite worldwide vaccination programs, whooping cough, also known as pertussis, remains a controversial global public health problem. Vaccination is the most effective control measure against this disease [1]. Whole-cell pertussis vaccines (wPVs) have been available since the mid-twentieth century and have proven highly effective [2]. However, owing to high reactogenicity following immunization and inconsistencies in vaccine production, wPVs have been replaced by acellular vaccines (aPVs), which have improved safety profiles [1]. The protective immune responses induced by these parenterally administered vaccines involve both systemic cellular and humoral immune responses but fail to induce mucosal immune responses [2, 3]. This is a critical limitation, since *Bordetella* (*B.) pertussis* is a mucosal pathogen, the induction of mucosal immunological defense, in addition to appropriate systemic immune responses, provides more effective protection [4].

Efforts to investigate mucosal vaccination routes against pertussis have been conducted in both humans and mice, but none have progressed to widespread implementation. Nasal administration of aPV combined with *Escherichia (E.) coli* heat-labile enterotoxin (LT) enhanced antigen-specific serum IgG, sIgA, and local/systemic T cell responses, leading to faster clearance of *B. pertussis* from the lungs after challenge. A fusion protein containing two copies of the S1 subunit of pertussis toxin fused to cholera toxin (CT) induced anti-pertussis toxin serum IgG and mucosal IgA, reducing lung bacterial counts in vaccinated mice compared with non-vaccinated mice [5]. Although adjuvants such as CT and LT are necessary to elicit strong immune responses, concerns about potential side effects, such as Bell’s palsy associated with CT and LT, limit their use in humans [6].

Another efficient approach for mucosal vaccine development strategies is the targeting of mucosal antigen-presenting cells (APCs) for appropriate processing of antigens that lead to specific T and B cell activation. Initiation of antigen-specific immune responses occurs in organized mucosa-associated lymphoid tissues (MALTs). MALTs play pivotal roles in mucosal immunological responses. MALT is covered by follicle-associated epithelium (FAE), which contains M cells, key antigen-sampling cells responsible for delivering antigens to the underlying APCs. Efficient delivery of antigens to M cells is essential for mucosal vaccinations [7–9]. A high-affinity ligand that binds to M cells is *Clostridium perfringens* enterotoxin (CPE). The C-terminal fragment of CPE (C-CPE, residues 184–319) binds to Claudin-4, a highly expressed protein on FAE and M cells. Claudin-4 is a tight junction protein identified as an M cell endocytosis receptor [10, 11]. Claudin targeting has been successfully used to elicit mucosal and systemic immune responses [10]. Nasal immunization with a fusion protein of ovalbumin with C-CPE (OVA-C-CPE) enhances the production of OVA-specific serum IgG and nasal sIgA [12]. Recently, intranasal administration of C-CPE fused with the receptor binding domain (RBD) of SARS-CoV-2 induced robust mucosal and systemic anti-SARS-CoV-2 neutralizing antibody responses, as well as systemic T cell responses against the virus [13]. Additionally, recombinant influenza hemagglutinin (HA) combined with the C-CPE peptide has been shown to induce increased mucosal IgA responses [14]. These results indicate that claudin-4 targeting may be a potent approach for enhancing mucosal immunity by facilitating the uptake of antigens across mucosal surfaces. We conducted an in silico analysis of a novel chimeric protein (F1S1-C-CPE) as a candidate vaccine against pertussis [15]. This novel protein comprises the S1 subunit of pertussis toxin and the type 1 immunodominant domain of filamentous hemagglutinin (F1) as its antigenic component. The C-CPE served as the delivery system. Our computational findings indicate that F1S1-C-CPE is a stable protein that efficiently binds to Claudin-4 [15].

Multiepitope vaccines, which incorporate carefully selected epitopes to activate immune cells, represent a promising strategy. These vaccines offer the advantage of customization, enabling the inclusion of highly immunogenic regions from the pathogen’s antigens [16–18]. In this study, we utilized a reverse vaccinology approach to design a multiepitope vaccine targeting *B. pertussis* on the basis of the F1S1-C-CPE protein. Advanced immunoinformatics tools were used to identify epitopes that are immunogenic and safe, ensuring broad coverage and minimal adverse reactions. By combining these elements, this research aimed to evaluate the structural, immunological, and biological properties of novel vaccine candidates.

## Materials and methods

### Sequence retrieval and evaluation of antigenicity

The amino acid sequences of F1 and S1, as well as C-CPE from *Clostridium perfringens*, were retrieved from UniProt (https://www.uniprot.org/). The VaxiJen v2.0 server (http://www.ddg-pharmfac.net/Vaxijen/VaxiJen/VaxiJen.html) [19] was used to predict the antigenicity of the F1 and S1 proteins.

### Ethics statement

The research was approved by the Ethics Committee of the Pasteur Institute of Iran (IR.PII.AEC.1403.017).

### T lymphocyte epitope prediction

#### Cytotoxic T lymphocyte (CTL) epitope prediction

For the prediction of 9-mer CTL epitopes, the amino acid sequences of S1 and F1 were submitted to the proteasomal cleavage/TAP transport/MHC class I combined predictor on the IEDB platform (http://tools.iedb.org/processing/). Additionally, each CTL epitope was further predicted for binding to other HLA class I alleles via the immune epitope consensus method available through the Immune Epitope Database (IEDB) server (https://tools.iedb.org/mhci/). Epitopes with a percentile rank below 5 were designated good binders, as lower scores indicate higher affinity, and those with an IC50 < 50 nM were selected for further analysis. These selected epitopes were then assessed for potential toxic properties via the ToxinPred server (http://crdd.osdd.net/raghava/toxinpred/), and their immunogenicity was estimated via the IEDB MHC-I immunogenicity tool (http://tools.iedb.org/immunogenicity/).

#### Prediction of helper T lymphocyte (HTL) epitopes

The 15-mer HTL epitopes from S1 and F1 were predicted via the NetMHCIIpan 4.1 EL approach for MHC II binding via IEDB tools (https://tools.iedb.org/mhcii/). The species/locus was set as Human/HLA-DP, HLA-DQ, or HLA-DR. Conserved immune-dominant peptides with a percentile rank of less than 5, indicating strong binding to MHC II molecules, were selected for further analysis. The selected HTL epitopes were subsequently evaluated for allergenicity via the AlgPred server (https://webs.iiitd.edu.in/raghava/algpred/submission.html). Additionally, these epitopes were screened for antigenicity and toxicity via the VaxiJen v2.0 and ToxinPred servers, respectively. The selected HTL epitopes were then submitted to three online tools to assess their potential to induce interferon-gamma (IFN-γ) and interleukin-4 (IL-4), namely, the IFN epitope server (https://webs.iiitd.edu.in/raghava/ifnepitope/index.php) and the IL4pred server (https://webs.iiitd.edu.in/raghava/il4pred/predict.php).

### B cell epitope prediction

For the prediction of linear B cell epitopes, the amino acid sequences of the S1 subunit and F1 were analyzed via the BepiPred-2.0 tool on the IEDB server (http://tools.iedb.org/bcell/). Additionally, potent conformational B cell epitopes (non-linear B cell epitopes) in these antigens were evaluated via ElliPro (http://tools.iedb.org/ellipro/) on the IEDB server. ElliPro estimates conformational epitopes on the basis of the 3D structure of the protein, considering solvent accessibility and flexibility. The allergenicity, antigenicity, and toxicity of the selected epitopes were further evaluated via the AllerTOP v.2.0 (https://ddg-pharmfac.net/AllerTOP/), VaxiJen v2.0, and ToxinPred servers, respectively. The IgPred server (https://webs.iiitd.edu.in/raghava/igpred/index.html) was employed to predict antibody class-specific B cell epitopes.

### Analysis of cross-reactivity

To assess whether the predicted epitopes matched sequences within the human proteome, the PIR-International Protein Sequence Database (https://research.bioinformatics.udel.edu/peptidematch/batchpeptidematch.jsp) was utilized.

### Population coverage of CTL and HTL epitopes

The IEDB population coverage analysis tool (https://tools.iedb.org/population) was used to analyze the population coverage of the chosen CTL and HTL epitopes and their corresponding HLA alleles. The default parameters were used, and the coverage was checked against the combination of HLA class I- and HLA class II-binding alleles.

### Multiepitope vaccine construct and structural analysis

The final screened CTL, HTL, and linear B cell (LBL) epitopes were linked together by glycine-proline-rich (GPGPG) linkers. In accordance with our previous study, the C-terminus of the construct was appended with C-CPE, which contains 136 amino acids and serves as a delivery system to target claudin-4 receptors via a G4S linker [15]. The secondary structure of the vaccine construct was obtained via the PSIPRED 4.0 server (http://bioinf.cs.ucl.ac.uk/psipred/) and the RaptorX Property server (http://raptorxuchicago.edu/StructurePropertyPred/predict/). The RaptorX Property server was used to calculate the secondary structure (SS), disordered regions (DISO), and solvent accessibility (ACC) [8]. The tertiary structure of the final multiepitope subunit vaccine was generated via the Robetta server (http://robetta.bakerlab.org/) with the RoseTTAFold method [20]. The server shows five modeled structures and their respective confidence scores to evaluate the quality of the predicted models. The most desirable three-dimensional structure modeled by the Robetta server with the best confidence was further refined via the GalaxyRefine server (http://galaxy.seoklab.org/cgibin/submit.cgi?type=REFINE), leading to an increase in the number of residues in the favored region and the generation of five refined models. The stability of the tertiary structures was validated via the ProSA web server (https://prosa.services.came.sbg.ac.at/prosa.php) on the basis of the Z score. The ERRAT web server (http://services.mbi.ucla.edu/ERRAT/3) was also employed to compute the non-bonded atom‒atom interactions. Finally, the Ramachandran plot was retrieved from the PROCHECK server (https://servicesn.mbi.ucla.edu/PROCHECK/) to evaluate the stereochemical qualities of the modeled structure.

### Prediction of physicochemical, immunological, and biological properties

The ProtParam tool from the ExPASy server (https://web.expasy.org/protparam/) was used to assess various physicochemical properties of the final multiepitope construct. Furthermore, the vaccine candidate construct was also screened for transmembrane helices via the TMHMM server v2.0. Finally, the solubility of the vaccine construct was evaluated via the SOLpro server (http://scratch.proteomics.ics.uci.edu/). In addition, the construct was investigated via Vaxijen v2.0 and cross-checked with the ANTIGENpro server (http://scratch.proteomics.ics.uci.edu/explanation.html#ANTIGENpro). To screen for potential causes of allergic reactions, we used the AllerTOP v2.0 and AllergenFP v1.0 (https://ddg-pharmfac.net/AllergenFP/) servers to assess the allergenicity of the vaccine protein. Next, the toxicity of the vaccine construct was estimated via the ToxinPred server.

### C-CPE immunodominance and processing analysis

NetChop 3.1 server (https://services.healthtech.dtu.dk/service.php?NetChop-3.1) was employed to analyze cleavage patterns across the full mF1S1-C-CPE construct, with emphasis on linker regions and domain junctions. Antigenicity of C-CPE was assessed using VaxiJen v2.0 (threshold = 0.4), with allergenicity predictions via AllerTOP v2.0 and AlgPred. IEDB MHC I/II binding tools analyzed C-CPE-derived epitopes using same parameters as F1/S1 (percentile rank < 5 for HLA alleles). IFN epitope and IL4pred servers assessed C-CPE’s capacity to induce IFN-γ/IL-4 responses.

### Molecular docking simulation of peptide‒MHC binding

The GalaxyPepDock web server (http://galaxy.seoklab.org/cgi-bin/submit.cgi?type=REFINE) was used to conduct protein‒peptide docking between the selected MHC class I and MHC class II PDB files and their corresponding peptide epitopes. The ClusPro 2.0 web server (https://cluspro.org/login.php) was used to conduct protein–protein docking between the selected MHC class I and MHC class II PDB files and their corresponding peptide epitopes in the full polypeptide construct (mF1S1-C-CPE). Upon completion of the docking process, the top model complex was refined via GalaxyRefineComplex (http://galaxy.seoklab.org/cgi-bin/submit.cgi?type=COMPLEX). The Gibbs free energy (ΔG) and dissociation constant (Kd) for each complex were subsequently calculated via the PRODIGY server (PROtein binDIng enerGY prediction) (https://rascar.science.uu.nl/prodigy/), a tool designed to predict the binding affinity of protein‒protein complexes on the basis of their 3D structure.

### Molecular docking simulation of mF1-S1-C-CPE with TLRs

The tertiary structure of human TLR2 and TLR4 was obtained from Protein Data Bank (PDB: 6NIG and 8WTA). Docking studies were carried out using ClusPro 2.0 webserver to find the interaction pattern of the full polypeptide construct (mF1S1-C-CPE) with TLR2 and TLR4. the top model complex was refined using GalaxyRefineComplex and evaluated for ΔG and Kd using the PRODIGY server.

#### Molecular dynamics (MD) simulations

MD Simulations of mF1S1-C-CPE, mF1S1-C-CPE/TLR2 and mF1S1-C-CPE/TLR4 complexes were performed in GROMACS 2022.6 using the all-atom optimized potentials for liquid simulations (OPLS-AA) force field [21]. Systems were solvated with the SPC water model in a cubic box and neutralized with Cl⁻/Na⁺ counterions. Energy minimization employed a 10-kJ/mol/nm tolerance threshold across 500,000 steps, followed by NVT/NPT ensemble equilibration via the Verlet integrator (0.01 ps timestep). Production runs used a 2-fs timestep for 100 ns at 300 K under periodic boundary conditions, with long-range electrostatics handled by Particle Mesh Ewald (1.2 nm Coulomb cutoff). Conformational stability was assessed through the root mean square deviation (RMSD) of backbone atoms, root mean square fluctuation (RMSF) for residue-specific flexibility, and radius of gyration (Rg) for each trajectory.

### Codon optimization and in silico cloning of the vaccine

The VectorBuilder server (available at https://en.vectorbuilder.com/tool/codon-optimization/) was utilized for codon optimization of the vaccine construct, with *Escherichia*** (***E.) coli* K12 chosen as the source organism. The codon adaptation index (CAI) and GC content of the optimized sequence were analyzed to assess expression levels in the host. Additionally, SnapGene software (https://www.snapgene.com/free-trial/) was employed for in silico cloning, allowing the optimized final vaccine candidate sequence to be inserted into the pET21a vector.

## Results

### Protein sequences and their antigenicity

The FASTA formats of the F1 protein (UniProt ID: P12255) and the S1 subunit of pertussis toxin (PT) (UniProt ID: P04977) from *B. pertussis*, as well as the C-CPE (UniProt ID: P01558) from *C. perfringens*, were retrieved from UniProt. Additionally, the antigenicity scores of the F1 and S1 proteins were obtained via the Vaxijen v2.0 server, with scores of 0.8411 and 0.5718, respectively. The threshold of the server was set at 0.4. Furthermore, the PDB files for F1 (PDB ID: 1RWR) and the S1 subunit of PT (PDB ID: 1BCP) were retrieved from the PDB databank.

### T cell epitopes of F1 and S1 antigens

Computational prediction of CTL and HTL epitopes for proteins S1 and F1 was performed via MHC class I- and II-binding algorithms hosted by the IEDB Analysis Resource. The CTL epitopes were determined via the Consensus method, whereas the HTL epitopes were identified via the NetMHCIIpan 4.1 EL method. First, 730 and 12,744 CTL epitopes were identified from the S1 and F1 proteins, respectively (Supplementary Tables S1 and S2), on the basis of the reference sets of all HLA alleles. These epitopes were subsequently screened according to their high binding affinity scores (IC50 and percentile rank), antigenicity, toxicity, and immunogenicity (Table 1).

**Table 1:** CTL epitopes from S1 (upper part) and F1(lower part) antigens

Peptide	HLA allele	Length	Percentile rank	IC_50_	Toxicity	Immunogenicity score	Antigenicity score
AGRGTGHFI^a^	HLA-A^a^30:01	9	1.5	28.41	Non-toxic	0.2163	0.4113
CTRAIRQTA	HLA-A^a^30:01	9	0.6	16.63	Non-toxic	0.15941	0.7997
EVRADNNFY	HLA-A^a^26:01	9	0.13	46.86	Non-toxic	0.13462	1.0706
DTYGDNAGR	HLA-A^a^68:01	9	0.145	12.11	Non-toxic	0.10123	2.089
NVLDHLTGR	HLA-A^a^33:01 HLA-A^a^68:01	9	0.55	49.52	Non-toxic	0.09234	0.5681
QTRANPNPY	HLA-A^a^30:01	9	2.6	32.28	Non-toxic	0.02749	0.8004
RAGEAMVLV	HLA-A^a^02:06	9	1.98	33.77	Non-toxic	0.01291	0.7949
IEAGGNARL^a^	HLA-B^a^40:01	9	0.175	16.87	Non-toxic	0.13697	1.6518
HTLESAEGR	HLA-A^a^68:01	9	0.415	16.29	Non-toxic	0.07718	1.5158
YIWGLYPTY	HLA-B^a^35:01	9	0.7	8.04	Non-toxic	0.10504	1.4533
EAYGEATRR	HLA-A^a^68:01	9	0.205	14.91	Non-toxic	0.23023	1.1888
RYEYIWGLY	HLA-A^a^30:02	9	0.31	15.15	Non-toxic	0.38901	1.0929
AEHDATLTL	HLA-B^a^40:01		0.11	4.6	Non-toxic	0.12078	0.8581
KPAPTAPPM	HLA-B^a^07:02 HLA-A^a^02:06	9	0.2	6.79	Non-toxic	0.06033	0.8117
EDMHLDAPR	HLA-A^a^68:01	9	0.755	48.32	Non-toxic	0.01217	0.7701
ATRRVHDQL	HLA-A^a^30:01	9	1.7	42.49	Non-toxic	0.09057	0.749
MAVQAVEAY	HLA-B^a^15:01 HLA-B^a^58:01 HLA-B^a^35:01	9	0.1	2.97	Non-toxic	0.08116	0.6478
FAADLRTVY	HLA-B^a^35:01	9	0.2	4.09	Non-toxic	0.12982	0.6379
RLTAAVALL	HLA-A^a^02:03	9	1.075	27.37	Non-toxic	0.15547	0.4058

^a^Selected peptides in the final construct are those underlined

Additionally, 6885 and 12,879 HTL epitopes (15-mers) were identified from the S1 and F1 proteins, respectively (Supplementary Tables S3 and S4), on the basis of the reference sets of all HLA alleles. These epitopes were subsequently screened on the basis of the IC50 and percentile rank; antigenicity; toxicity; allergenicity; and the potential to induce IFN-γ and IL-4 (Table 2).

**Table 2:** HTL epitopes of S1 (upper part) and F1(lower part) antigens

Allele	Start	End	Length	Peptide	IC_50_	Rank	Toxicity	Allergenicity	IFN-γ	IL-4	Antigenicity
HLA-DQA1^a^01:02 DQB1^a^06:02 HLA-DRB1^a^11:01	220	234	15	SIVGTLVRMAPVIGA	21.9	0.19	Non-toxic	Non-allergen	+	Inducer	0.4505
HLA-DQA1^a^01:02 DQB1^a^06:02	218	232	15	VASIVGTLVRMAPVI	20.7	0.16	Non-toxic	Non-allergen	+	Inducer	0.3061
HLA-DRB1^a^11:01	219	233	15	ASIVGTLVRMAPVIG	44.4	4.6	Non-toxic	Non-allergen	+	Inducer	0.4199
HLA-DRB1^a^13:02	209	223	15	IEVGKDLYLNAGARK	5.2	0.41	Non-toxic	Non-allergen	−	Inducer	0.68
HLA-DRB3^a^01:01	293	307	15	LDYLLDQNRYEYIWG	27.6	1.2	Non-toxic	Non-allergen	+	Inducer	0.1912
HLA-DRB3^a^01:01	414	428	15	GIIQEFAADLRTVYA	29.6	1.3	Non-toxic	Non-allergen	+	Inducer	0.1891
HLA-DRB3^a^01:01	294	308	15	DYLLDQNRYEYIWGL	43.5	2	Non-toxic	Non-allergen	+	Inducer	0.3298
HLA-DRB1^a^13:02	100	114	15	KAQNITNKRAALIEA	15.7	2.1	Non-toxic	Non-allergen	+	Inducer	0.4100
HLA-DRB3^a^01:01	100.5	114.5	15	LDYLLDQNRYEYIWG	23.65	2.5	Non-toxic	Non-allergen	+	Inducer	0.1912
HLA-DRB1^a^11:01	126	140	15	LLNKLGRIRAGEDMH	22.6	2.3	Non-toxic	Non-allergen	−	Inducer	0.0534
HLA-DRB1^a^13:02	98	112	15	TVKAQNITNKRAALI	17.5	2.4	Non-toxic	Non-allergen	+	Inducer	0.6475
HLA-DRB1^a^07:01	250	264	15	RSVVRTVSAMEYFKT	32.2	3.9	Non-toxic	Non-allergen	+	Inducer	− 0.3914
HLA-DRB1^a^09:01	250	264	15	RSVVRTVSAMEYFKT	45.2	3.9	Non-toxic	Non-allergen	+	Inducer	− 0.3915

^a^Selected peptides in the final construct are those underlined

### *B cell epitope prediction from S1 and F1 antigens*

The BepiPred-2.0 tool from the IEDB server was used for linear B cell epitope mapping. As a result, 12 and 16 linear epitopes were identified from the S1 and F1 proteins, respectively (Supplementary Tables S5 and S6). Following the evaluation of antigenicity, allergenicity, toxicity, and immunoglobulin production capacity, one epitope was chosen from the S1 epitopes, and the other was chosen from the F1 epitopes (Table 3). Additionally, with the use of ElliPro software, one conformational B cell epitope, A: D30, A: N31, A: Y178, A: T179, A: S180, and A: R181, was identified for the S1 antigen. Notably, the sequences A: Y178, A: T179, A: S180, and A: R181 of the S1 conformational B cell epitope lie within the selected LBL epitope of this antigen (highlighted in gray in Table 3). Whole sequences of this conformational epitope was not included in the final vaccine construct. The total numbers of initially predicted CTL, HTL, and B cell epitopes, as well as the numbers after filtration, are presented in Supplementary Table S7.

**Table 3:** B cell epitopes of S1 and F1 antigens

Antigen name	Start	End	Peptide	Length	Toxicity	Allergenicity	Antibody class prediction/score	Antigenicity
S1	28	36	VTSPAWADD	9	Non-toxic	None	Non-Epi	0.2688
	44	52	YDSRPPEDV	9	Non-toxic	None	Non-Epi	0.6006
	105	117	AVEAERAGRGTGH	13	Non-toxic	None	Non-Epi	1.8023
	139	151	FEYVDTYGDNAGR	13	Non-toxic	None	Non-Epi	1.1297
	184	217	NGITGETTTTEYSNARYVSQQTRANPNPYTSRRS	34	Non-toxic	None	IgG/1.194	1.0259
F1	43	50	TTEIETGN	8	Non-toxic	None	Non-Epi	1.4583
	60	70	ENIDNKQAIVV	11	Non-toxic	None	Non-Epi	0.2878
	86	91	EANALL	6	Non-toxic	None	Non-Epi	−0.238
	104	112	ITNKRAALI	9	Non-toxic	None	Non-Epi	−0.2878
	130	136	LGRIRAG	7	Non-toxic	None	Non-Epi	−0.1518
	260	285	EYFKTPLPVSLTALDNRAGLSPATWN	26	Non-toxic	None	IgG/1.071	0.4516
	298	302	DQNRY	5	Non-toxic	None	Non-Epi	N/A

Selected peptides in the final construct are those underlined

### Population coverage

To design the final multiepitope construct targeting the S1 and F1 proteins, nine CTL epitopes (four from protein S1 and five from protein F1) and five HTL epitopes (two from protein S1 and three from protein F1) were selected. These epitopes, along with their corresponding HLA alleles, were used to estimate population coverage in combination with MHC class I and II molecules. The selected epitopes demonstrated an average population coverage of 65.58% across the regions listed in Table 4. Additionally, the coverage for specific regions is also detailed within the table. Notably, the value of 62.56% represents the worldwide population coverage, as directly calculated by the IEDB server using comprehensive data on HLA allele frequencies from global populations.

**Table 4:** Population coverage of multi-epitope vaccine

Population/area	Class combined		
	Coverage	Average hit	pc90^c^
Central Africa	84.81%	1.55	0.66
China	63.19%	0.96	0.27
Europe	67.4%	1.1	0.31
Iran	58.19%	0.9	0.24
Oceania	63.27%	0.92	0.27
South Asia	59.82%	1.0	0.25
Southeast Asia	67.66%	1.01	0.31
USA	63.33%	1.05	0.27
World	62.56%	1.0	0.27
Average	65.58	1.05	0.32
Standard deviation	7.38	0.18	0.12

### Multiepitope vaccine candidate construction

The final multiepitope vaccine candidate comprises a 440-amino acid protein sequence structured as follows: 9 CTL epitopes (bold), 5 helper HTL epitopes *(*underlined), and 2 LBL epitopes (red text), all connected via GPGPG linkers. To enhance mucosal targeting, a claudin-4-binding C-CPE fragment (136 amino acids, green text) was appended to the construct’s C-terminal end via a G4S linker.
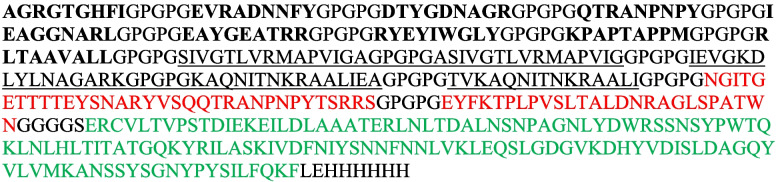


### Sequence and structural analysis and validation of the multiepitope construct

The secondary structure analysis of the 440 amino acid multiepitope construct revealed a composition of 17% α-helices, 17.5% β-sheets, and 65.5% coil structures.

Additionally, RaptorX property analysis of solvent accessibility predicted that 59% of the amino acid residues were exposed, 26% were moderately exposed, and 29% were buried. Furthermore, the software predicted that 2% of the positions were disordered. The results from the PIR server showed that the vaccine candidate construct has no sequence similarity to the human proteome, indicating that it is unlikely to induce cross-reactivity with self-proteins in the human body.

**Fig. 1:**
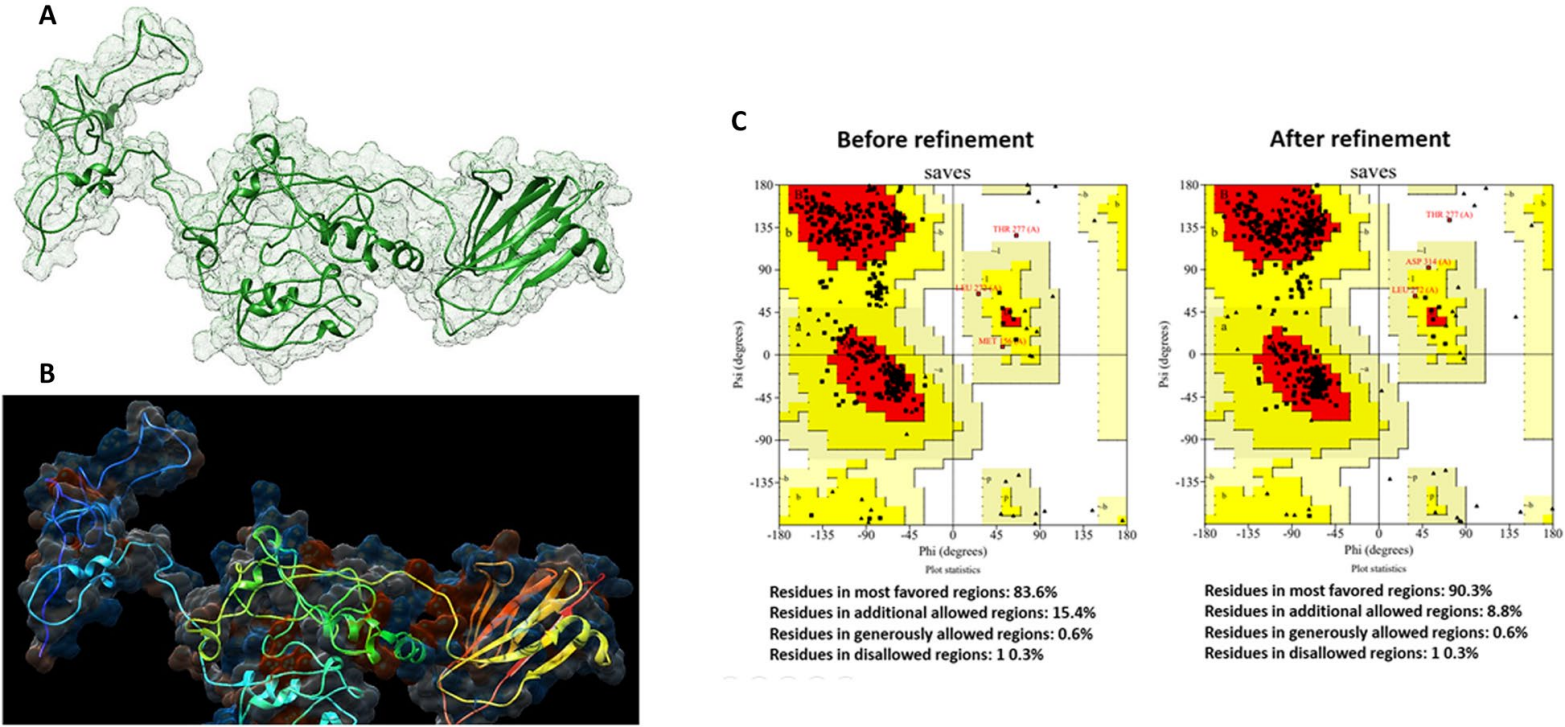
The 3D structure of the final construct of the vaccine candidate following refinement (**A**,** B**). Ramachandran plot for the final construct of the vaccine candidate before and after refinement (**C**)

The 3D structure of the final construct was modeled via the RoseTTAFold method on the Robetta server. The results indicated five structural models. The most desirable one (Fig. 1A, B), on the basis of their confidence scores, was further refined via the GalaxyRefine server. The refinement process improved the structural quality, as evidenced by a GDT-HA score of 0.9693, an RMSD of 0.359, a MolProbity score of 1.954, a clash score of 11.6, 0.9% poor rotamers, and 94.5% residues in the Ramachandran favored regions (Fig. 1C). The Ramachandran plot analysis, performed via the PROCHECK server, revealed notable improvements following refinement, with 90.3% of the residues located in the most favored regions, 8.8% in additionally allowed regions, 0.6% in generously allowed regions, and 0.3% in disallowed regions (Fig. 1C).

**Fig. 2:**
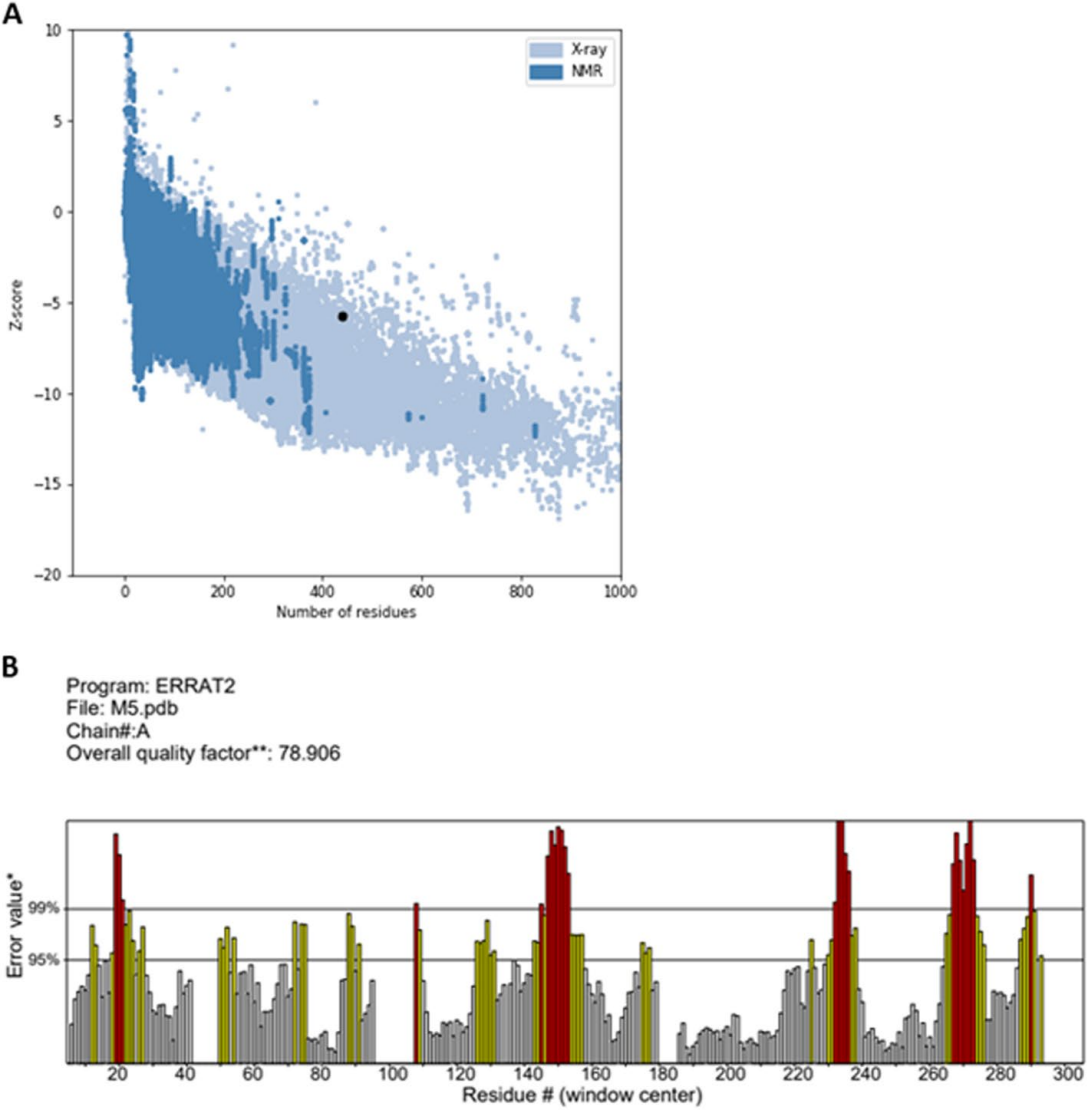
**A** ProSA-web server and **B** ERAT results showing that the final multiepitope construct is within the range of scores typically found for native protein conformations of similar size

To assess the structural quality and identify potential errors, ProSA-web and ERRAT analyses were conducted. The refined model had an overall quality factor of 78.9% in the ERRAT, whereas ProSA-web displayed a *Z*-score of − 5.42 for the vaccine candidate model (Fig. 2A, B). These results indicate that the final multiepitope construct falls within the score range typically observed for native protein conformations of similar size.

### Physicochemical and biological properties of the multiepitope construct

The results of the physicochemical and biological analyses of the multiepitope vaccine candidates are presented in Table 5. The ProtParm results indicated that the instability index (II) for the multiepitope construct was 27.19, suggesting that the vaccine will remain stable after expression. Additionally, its aliphatic index was predicted to be 70.14, which estimates the construct’s thermostability. The GRAVY score of − 0.471 indicates the polar nature of the vaccine construct. Furthermore, the SOLpro server showed high solubility for the multiepitope vaccine candidate, with a score of 0.755444. The construct also demonstrated strong antigenic properties, with scores of 0.8926 and 0.895885 obtained from VaxiJen v2.0 and ANTIGENpro, respectively.

**Table 5:** Physicochemical and biological features of the multi-epitope vaccine candidate

Properties	Assessment
Length	440 amino acids
Mw	45620.04 Da
pI	9.39
Instability index	27.19/stable
Aliphatic index	70.14
GRAVY index	− 0.471
Antigenicity	Antigen/0.8926 (Vaxijen v.2.0) Antigen/0.895885 (ANTIGENpro)
Allergenicity	Non-allergen
Toxicity	Non-toxic
Solubility	Soluble − 0.755444

The final construct was identified as non-allergenic and non-toxic on the AllerTOP v.2 and ToxinPred servers. Moreover, the results from the IgE epitope mapping tools in AlgPred 2.0 indicated that our construct does not contain any IgE-inducing epitopes, confirming its non-allergenic structure. Additionally, the vaccine candidate was assessed for the number of transmembrane helices via the TMHMM v2.0 server, which revealed that it does not contain any transmembrane helices.

### Conformational B cell epitopes in the multi-epitope vaccine construct

**Fig. 3:**
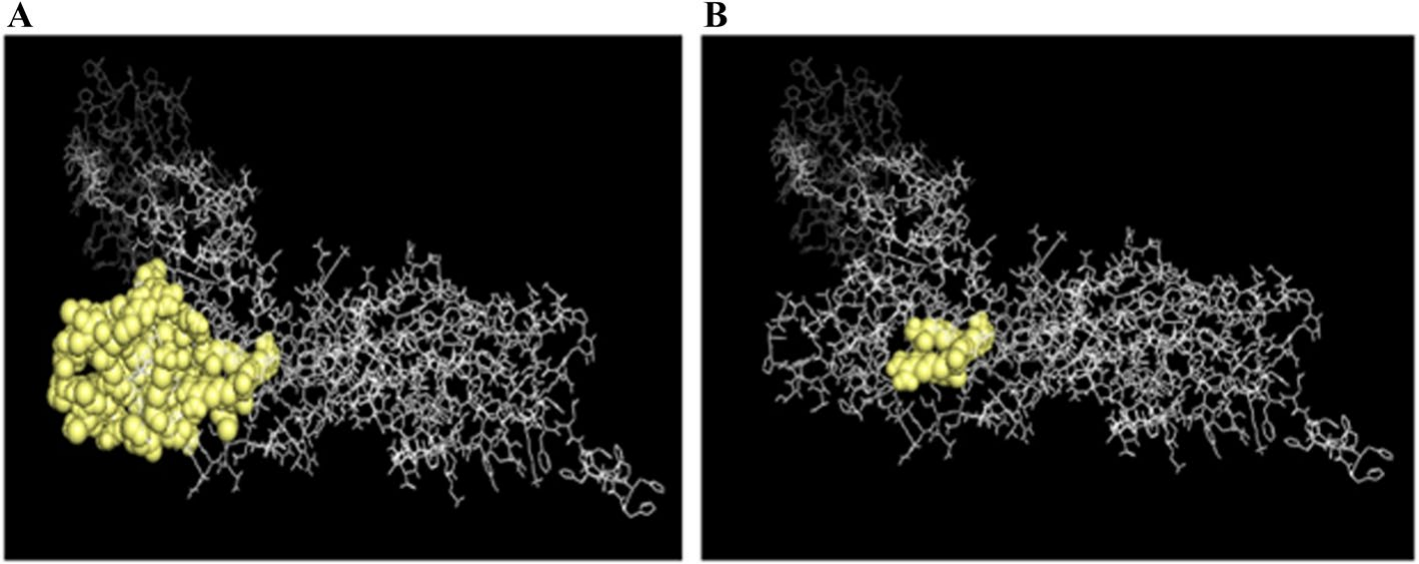
**A** Conformational B cell epitopes in the multi-epitope vaccine construct predicted by ElliPro are depicted as yellow spheres. **B** Key residues (A:Y255, A:T256, A:S257, and A:R258) from the original S1 epitopes as a contiguous patch

ElliPro analysis was performed on the refined 3D model of the vaccine construct to evaluate surface accessibility of B cell epitopes, particularly A:Y178, A:T179, A:S180, A:R181 residues from the original S1 epitopes. ElliPro identified several surface-exposed epitopes in the vaccine construct (Fig. 3A), including the aforementioned residues, which are depicted as yellow spheres in Fig. 3B. These residues form a contiguous patch (positions 227–291) maintain a similar topology to the original S1 epitopes, despite architectural differences in the vaccine’s folded structure. The retained accessibility of key residues suggests that glycine-rich linkers (GPGPG) and domain orientation can preserve critical epitope conformations.

### Processing of C-CPE and immunodominance profile

NetChop predictions revealed several cleavage sites in the full construct, though most of cleavages were in linkers (Supplementary Table S8), suggesting efficient release of embedded epitopes. The fusion of C-CPE to the C-terminus of the construct was mediated by a flexible G4S linker, which is commonly used to minimize steric interference in folding of fused domains. C-CPE displayed moderate antigenicity with a VaxiJen score of 0.52), while being non-allergenic (AllerTOP score: 0.89) and non-toxic. Additionally, 498 HTL epitopes (15-mer; Supplementary Table S9) and 2472 CTL epitopes (9-mer; Supplementary Table S10) were identified from the C-CPE protein, based on reference sets of all HLA alleles. Only 19 predicted C-derived CTL epitopes showed strong binding to HLA-A/B alleles (IC50 < 50 nM). None of the C-derived CTL or HTL epitopes exhibited positive immunogenicity scores. Overall, both the number and binding affinity of predicted epitopes from the C protein were considerably lower than those derived from the S1 and F1 antigens, suggesting a reduced likelihood of the C protein outcompeting target epitopes during antigen processing.

### Molecular docking analysis of T cell epitopes with their respective HLAs

**Fig. 4:**
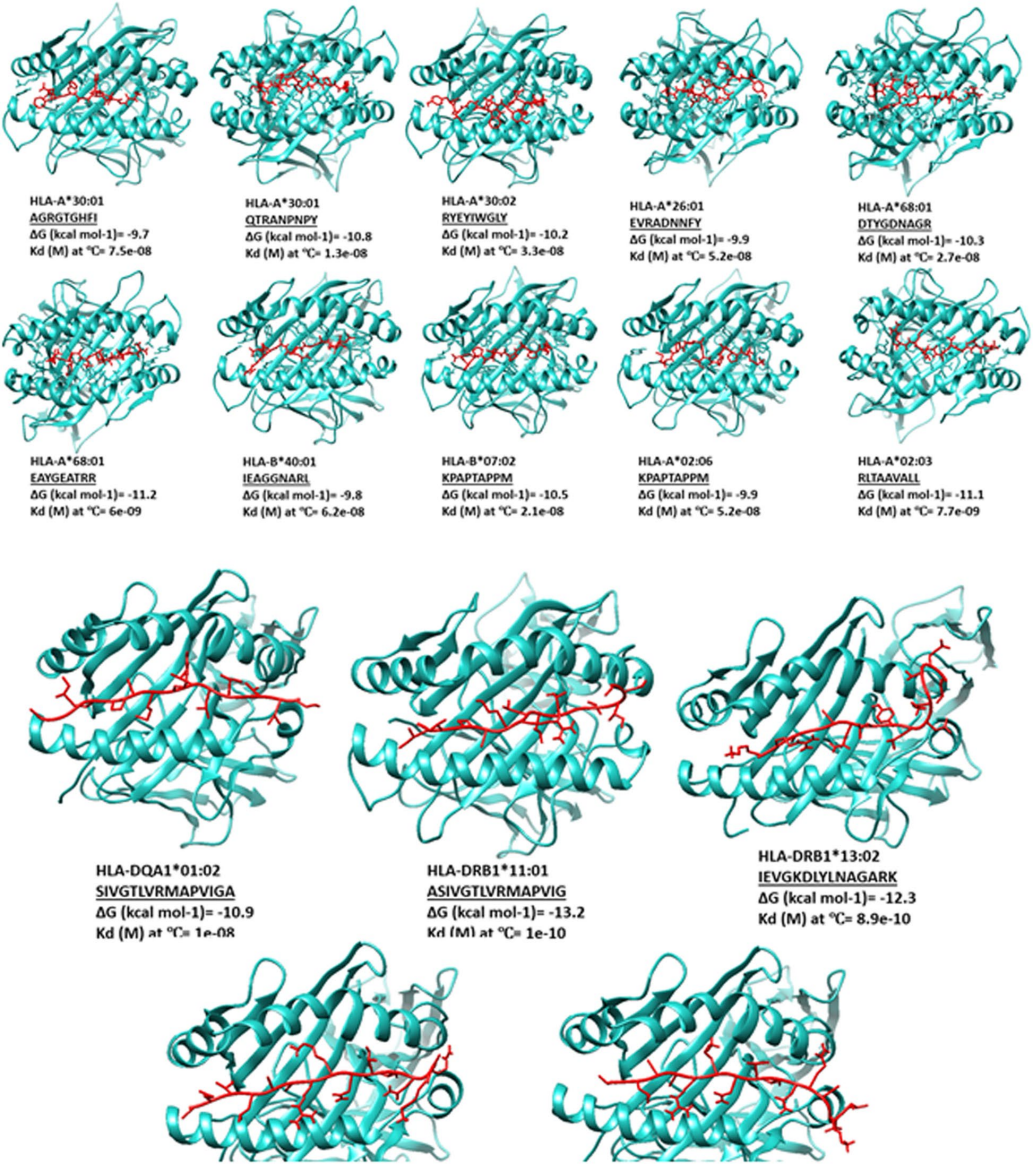
Molecular docking between the peptide epitopes and selected HLAs. HLA alleles and epitopes represent blue ribbon and red stick structures, respectively

The docked complexes obtained from the GalaxyPepDock server were analyzed via the PRODIGY server to measure the Gibbs free energy (ΔG) and the dissociation constant (Kd) for the peptide epitope-HLA the full polypeptide construct (mF1S1-C-CPE)-HLA complexes. The results indicated that the grooves of the respective HLA alleles fit most of the residues of the epitopes, facilitating binding through interactions between the R groups of the side chains and the pockets located on the floor of the HLA molecules. According to the PRODIGY results, the ΔG and Kd values confirmed the high-affinity binding patterns between the peptide and the corresponding HLA molecules (Fig. 4). The results of the full multiepitope polypeptide construct were compared with the results from individual epitope docking against the same panel of MHC (HLA) alleles (Table 6). For most HLA alleles, the full polypeptide construct demonstrated ΔG and Kd values indicative of high-affinity binding, comparable to or in some cases stronger than those observed for the individual epitope-HLA complexes. In a few instances (e.g., EVRADNNFY with HLA-A*26:01, DTYGDNAGR* with *HLA-A*68:01), the full construct showed slightly less favorable binding than the isolated epitope, but the affinities remained within a high-affinity range, supporting the overall structural compatibility of the multiepitope format.

**Table 6:** Comparison of binding affinities of full polypeptide construct (mF1S1-C-CPE) and individual epitopes with MHC alleles based on molecular docking

Complex	Epitopes within mF1S1-C-CPE		Individual epitopes	
	ΔG (kcal mol^−1^)	*K*_d_ (M) at ℃	ΔG (kcal mol^−1^)	*K*_d_ (M) at ℃
HLA-A*30:01 AGRGTGHFI	− 13.2	2.10E − 10	− 9.7	7.50E − 08
HLA-A*30:01 QTRANPNPY	− 13.2	1.90E − 10	− 10.8	1.30E − 08
HLA-A*26:01 EVRADNNFY	− 8.8	3.20E − 07	− 9.9	5.20E − 08
HLA-A*30:02 RYEYIWGLY	− 16.1	1.50E − 12	− 10.2	3.30E − 08
HLA-A*68:01 DTYGDNAGR	− 8.9	3.90E − 07	− 10.3	2.70E − 08
HLA-A*68:01 EAYGEATRR	− 8.7	2.90E − 08	− 11.2	6.00E − 09
HLA-B*40:01 IEAGGNARL	− 9.9	5.40E − 08	− 9.8	6.20E − 08
HLA-B*07:02 KPAPTAPPM	− 12.5	6.90E − 10	− 10.5	2.10E − 08
HLA-A*02:06 KPAPTAPPM	− 14.7	1.80E − 11	− 9.9	5.20E − 08
HLA-A*02:03 RLTAAVALL	− 9.1	2.00E − 07	− 11.1	7.70E − 09
HLA-DQA1*01:02 SIVGTLVRMAPVIGA	− 8.6	4.90E − 08	− 10.9	1.00E − 09
HLA-DRB1*11:01 ASIVGTLVRMAPVIG	− 8.9	3.10E − 08	− 13.2	1.00E − 10
HLA-DRB1*13:02 IEVGKDLYLNAGARK	− 8.4	6.70E − 08	− 12.3	8.90E − 10
HLA-DRB1*13:02 KAQNITNKRAALIEA	− 7.2	4.80E − 07	− 10.8	1.10E − 08
HLA-DRB1*13:02 TVKAQNITNKRAALI	− 7.4	3.50E − 08	− 11.7	2.40E − 09

*indicates a specific allele or group of alleles within the HLA locus

### Molecular docking analysis of mF1-S1-C-CPE with TLRs

By docking the full-length vaccine model to the extracellular domains of TLR2 and TLR4, we evaluated the binding affinities and Kd to examine the binding and interaction between the vaccine construct and innate immune receptors. The mF1S1-C-CPE–TLR2 complex exhibited ΔG and Kd values of − 11.5 kcal/mol and 3.40 × 10⁻⁹ M, respectively, while the mF1S1-C-CPE –TLR4 complex showed stronger binding with ΔG = − 14.7 kcal/mol and Kd = 1.60 × 10⁻^11^ M. These results demonstrate high-affinity binding between m and both TLR2 and TLR4 molecules.

### Molecular dynamics simulation analysis

**Fig. 5:**
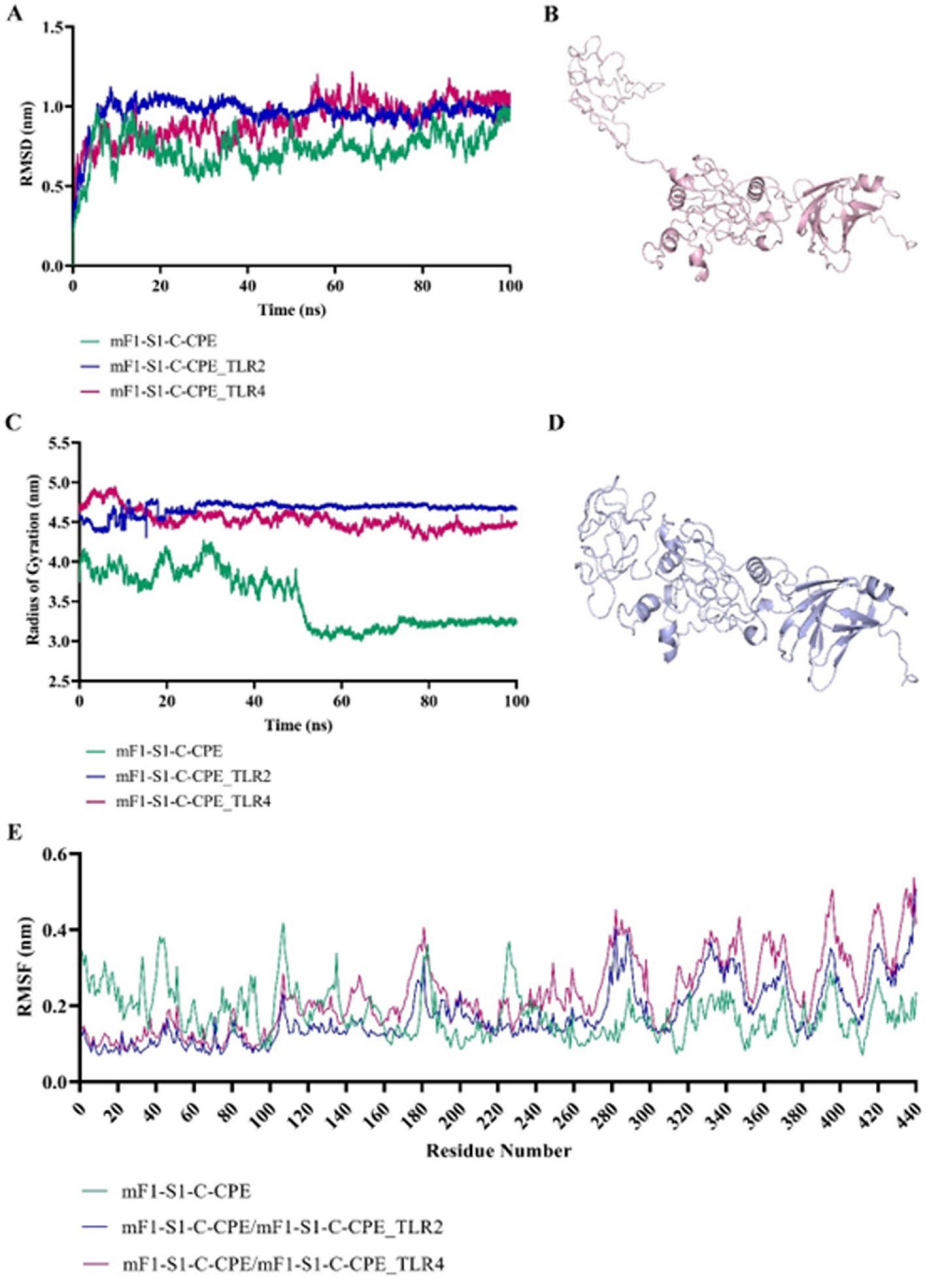
**A** RMSD plot of the backbone of mF1-S1-C-CPE, mF1-S1-C-CPE_TLR2, and mF1-S1-C-CPE_TLR4. **B** Cartoon views of predicted model mF1-S1-C-CPE. **C** Radius of Gyration plot of mF1-S1-C-CPE, mF1-S1-C-CPE_TLR2, and mF1-S1-C-CPE_TLR4. **D** Cartoon views of predicted model mF1-S1-C-CPE after MD simulation. **E** RMSF plot of mF1-S1-C-CPE residues

The structural stability and dynamic behavior of mF1-S1-C-CPE and its complexes with TLR2 and TLR4 were investigated through 100 ns all-atom MD simulations. The RMSD analysis (Fig. 5A) of the backbone atoms was performed to assess the conformational stability of the unbound mF1-S1-C-CPE and its complexes with TLR2 and TLR4. The mF1-S1-C-CPE system displayed a relatively stable RMSD profile with lower average deviations (~ 0.75 nm) throughout the simulation, indicating that the construct maintained a consistent conformation. Upon binding to TLR2 and TLR4, the RMSD increased slightly and exhibited greater fluctuations, particularly for mF1-S1-C-CPE_TLR4 (~ 1.0–1.2 nm). This suggests that binding to TLRs induced structural rearrangements and enhanced flexibility, possibly due to broader interface interactions or larger conformational adjustments upon receptor binding.

The compactness of the protein and its complexes was evaluated by the radius of gyration (Fig. 5C). The Rg profile of mF1-S1-C-CPE showed a gradual decrease over time from ~ 4.5 nm to ~ 3.2 nm, indicating an increase in structural compactness and possible folding during the simulation. In contrast, both mF1-S1-C-CPE_TLR2 and mF1-S1-C-CPE_TLR4 complexes maintained higher Rg values (~ 4.5–4.8 nm) with less variability, reflecting their relatively extended conformations and stable protein-receptor complex architecture. These results aligned with the expectation that larger complexes exhibit reduced structural compaction due to interfacial binding. Cartoon models (Fig. 5B, D) of mF1-S1-C-CPE before and after simulation illustrated the conformational refinement post-MD run. The structural compactness observed in the post-MD structure support the numerical findings from Rg and RMSD analyses.

The RMSF plot (Fig. 5E) was used to identify local flexibility across individual residues. The unbound mF1-S1-C-CPE construct demonstrated notable flexibility in loop and terminal regions (residues 30–60, 150–180, 250–270, and 400–440), which is typical of surface-exposed or disordered regions. Upon complexation with TLR2 and TLR4, the fluctuation profiles were dampened, particularly in mF1-S1-C-CPE_TLR2, suggesting stabilization of flexible segments upon receptor binding.

### *Reverse translation, codon optimization, and *in silico* cloning of the construct*

The length of the optimized codon sequence was 1344 nucleotides. The final vaccine construct presented a GC content of 62.29% when analyzed by VectorBuilder. This average GC content falls within the normal range of 30–70%. Our vaccine construct had a codon adaptation index (CAI) of 0.90, which was within the range of 0.8–1.0, indicating a strong possibility of good expression of the vaccine candidate in *E. coli*. Finally, the design of the recombinant vector was accomplished in silico by inserting the adapted codon sequences into the pET21a vector via SnapGene software. The raw sequence of the final vaccine candidate construct (excluding the start codon, stop codon, and enzymatic cleavage site) is presented below:

GTGGCCTGTGCAGGCCGCGGCACCGGCCATTTTATTGGCCCGGGCCCGGGCGAAGTGCGTGCGGATAACAATTTTTACGGACCGGGCCCGGGCGATACCTATGGTGATAACGCGGGCCGTGGCCCGGGTCCGGGCCAGACCCGTGCGAATCCGAATCCGTATGGCCCGGGTCCGGGCATTGAAGCGGGTGGCAATGCGCGCCTGGGCCCGGGCCCGGGCGAGGCGTATGGCGAAGCGACCCGCCGCGGTCCGGGCCCGGGCCGTTATGAATACATTTGGGGCCTGTATGGTCCAGGTCCGGGCAAGCCGGCGCCGACAGCGCCGCCGATGGGCCCGGGCCCGGGCCGCCTGACCGCGGCAGTTGCCCTGCTGGGCCCGGGCCCGGGCTCAATTGTGGGCACCCTGGTTCGTATGGCGCCGGTGATTGGCGCCGGCCCGGGTCCGGGCGCAAGCATTGTTGGCACCCTGGTGCGTATGGCGCCGGTGATTGGCGGTCCGGGTCCGGGCATTGAAGTGGGTAAAGATCTGTACCTGAATGCGGGCGCCCGCAAAGGCCCGGGCCCGGGCAAAGCGCAAAACATTACCAATAAACGTGCGGCGCTGATTGAAGCGGGCCCGGGCCCGGGCACCGTGAAAGCGCAGAACATTACCAATAAACGTGCGGCGCTGATTGGTCCGGGTCCGGGCAACGGTATTACCGGCGAAACCACCACCACCGAATATAGCAACGCCCGTTATGTTAGTCAGCAGACCCGCGCGAATCCGAACCCGTATACCAGCCGTCGTAGTGGCCCGGGCCCGGGCGAATACTTCAAAACCCCGCTGCCGGTGAGCCTGACCGCCCTGGATAATCGTGCTGGCCTGAGCCCGGCGACCTGGAATGGTGGCGGCGGCTCAGAACGTTGTGTGCTGACCGTTCCGAGCACCGACATTGAAAAAGAAATTCTGGATCTGGCAGCTGCCACCGAACGCCTGAATCTGACCGATGCCCTGAACAGCAACCCGGCGGGCAACCTGTATGATTGGCGCAGCAGCAACAGCTATCCGTGGACCCAGAAACTGAATCTTCATCTGACAATTACTGCGACCGGCCAGAAATACCGTATTCTGGCGTCCAAAATCGTTGACTTCAACATTTATTCGAACAATTTTAATAATCTGGTCAAATTAGAACAGAGCTTAGGCGATGGTGTGAAAGATCACTATGTGGATATTAGCCTGGATGCGGGCCAGTACGTGCTGGTGATGAAAGCAAACAGCTCATATAGCGGCAATTATCCGTATTCAATTCTGTTTCAGAAATTTCTGGAACATCATCATCATCATCATTAA

## Discussion

The need for an effective pertussis vaccine is underscored by the resurgence of whooping cough cases globally, despite the availability of traditional vaccines. These vaccines primarily induce systemic immune responses; however, mucosal immunity is particularly important because of *B. pertussis*’s primary residence in the upper respiratory tract, where it can evade systemic immune responses. Therefore, enhancing mucosal immunity is vital for effective protection against pertussis [7–9]. Previous studies on the F1S1 antigen, which comprises the S1 subunit of pertussis toxin and F1 from the FHA antigen, have demonstrated its potential in inducing both systemic and mucosal immunity when it is administered via subcutaneous or intranasal routes in BALB/c mice [22]. However, notably, immune responses are weaker following intranasal administration, highlighting the need for optimized delivery systems. By targeting claudin-4, C-CPE effectively delivers antigens to mucosa-associated lymphoid tissues and M cells and induces antigen-specific immune responses [23]. In a previous study, we applied computational analysis to a novel chimeric protein composed of C-CPE and F1S1 (F1S1-C-CPE). The results indicated that F1S1-C-CPE is a stable protein that efficiently binds to Claudin-4. Our findings suggest that fusing F1S1 antigens with C-CPE improves antigen uptake and stimulates both systemic and mucosal immune defenses [15]. In the present study, we aimed to develop a multiepitope vaccine incorporating F1S1 epitopes recognized by B and T cells, as well as C-CPE. The use of a multiepitope vaccine offers several advantages over traditional chimeric proteins. These vaccines enhance immune responses by including CTL, Th, and B cell epitopes, which stimulate both cellular and humoral immunity simultaneously. Additionally, they reduce unwanted components that may trigger pathological immune responses, making them safer than traditional or single-protein vaccines. The design of multiepitope vaccines also allows easy manipulation and customization on the basis of specific disease targets or populations [17, 18, 24].

In the present study, the prediction and validation of T cell and B cell epitopes via tools such as the IEDB and VaxiJen servers ensured that only the most immunogenic and non-allergenic epitopes were included in the final vaccine construct. This meticulous selection process reduces the risk of vaccine-related adverse effects, such as allergenicity and toxicity, which are concerns often raised in previous vaccine designs [19].

The secondary structural composition of our multiepitope construct shows a high coil content, indicating conformational flexibility, which is advantageous for epitope accessibility in vaccine constructs. Additionally, the solvent accessibility analysis revealed a significant proportion of exposed residues, suggesting potential surface accessibility for interactions. Moreover, the low disorder percentage implies a relatively stable structure. The structural quality assessment results indicate that the final multiepitope construct presents structural characteristics similar to those of native proteins, suggesting a high-quality model with minimal errors. This structural integrity is crucial for maintaining epitope presentation and stability, which are essential for inducing an effective immune response.

Compared with other multiepitope vaccines targeting *B. pertussis*, our design stands out for its focus on mucosal immunity. Roohparvar Basmenj et al. reported multiepitope constructs focused primarily on inducing systemic immune responses, with less emphasis on mucosal protection [25]. Although these approaches have successfully triggered robust T cell and B cell responses, the lack of mucosal engagement may limit their efficacy against respiratory pathogens. Our study complements these findings by highlighting the need for a dual-targeted approach that addresses both systemic and mucosal immunity.

The inclusion of both linear and conformational B cell epitopes in the final design is another strength of our approach. This ensures a more comprehensive immune response, as antibodies generated against linear epitopes may differ in function from those targeting conformational epitopes [16, 26]. Some studies have focused solely on linear B cell epitopes, potentially missing the broader spectrum of antibody responses that conformational epitopes can provide [27].

Moreover, in this multiepitope vaccine construct, T cell epitopes were incorporated alongside B cell epitopes. This strategic arrangement enhances the potential for a robust immune response by simultaneously presenting multiple targets to the immune system [28, 29]. The consecutive sequences of the HTL and CTL epitope peptides were fused together via a GPGPG linker. Additionally, the GGGGS flexible peptide linker was inserted before C-CPE to help maintain its functionality. This design aims to optimize the immune response by ensuring effective presentation and interaction of the epitopes with the immune system. Predicted cleavage sites in linkers facilitate APC-mediated proteolysis while preserving native epitope structure. The G4S linker’s flexibility minimizes domain interference, as evidenced by maintained structural stability during MD simulations (RMSD < 1.5 Å). Despite moderate antigenicity, C-CPE’s low HLA-binding affinity and absence of cytokine-inducing epitopes reduce competition with S1/F1 epitopes. This aligns with vaccine design principles prioritizing target antigen presentation over delivery system immunogenicity. The results of MHC docking analyses collectively indicate that the immunogenic epitopes retain their accessibility and high-affinity binding characteristics within the full polypeptide construct. The structural constraints imposed by the multiepitope assembly do not significantly compromise, and may in some cases enhance, the predicted immunogenicity at the MHC binding level. This supports the validity of using the multiepitope construct for further immunological evaluation and vaccine development.

Additionally, *B. pertussis* expresses several lipoproteins and other molecules that can engage TLR2 and TLR4, contributing to the immune response. The most likely TLR-interacting regions in our multi-epitope vaccine were the epitope regions that sequentially resemble natural TLR ligands**.** Analysis of ΔG and Kd values for the mF1S1-C-CPE/TLR2 and mF1S1-C-CPE/TLR4 complexes demonstrated strong binding affinity between mF1S1-C-CPE and both TLR2 and TLR4 receptors. Furthermore, the RMSD and Rg profiles from MD simulations of the mF1-S1-C-CPE construct and its interactions with TLR2 and TLR4 revealed that the unbound construct undergoes conformational tightening over time, indicative of structural stabilization. In contrast, the complexes with TLRs maintained more expanded and dynamically stable conformations, highlighting the structural influence of receptor binding. Overall, these findings support the structural integrity of the designed construct and demonstrate that interaction with immune receptors induces distinguishable dynamic signatures. Such differences may have functional implications for receptor recognition, signaling initiation, and vaccine or therapeutic design involving TLR engagement.

Population coverage analysis revealed that our vaccine construct offers substantial coverage across diverse geographic regions, including Central Africa, Southeast Asia, and Europe, with an average global coverage of 65.21%. This wide population coverage highlights the versatility of this vaccine in addressing global pertussis outbreaks, a consideration that is not consistently incorporated into other vaccine designs [30].

While this study employed advanced immunoinformatics and structural modeling tools to design and evaluate a multi-epitope vaccine candidate, several inherent limitations of the computational approach should be considered. First, in silico predictions of antigenicity, immunogenicity, and epitope-MHC binding are based on algorithmic models that, although validated, may not fully recapitulate the complexity of immune responses in biological systems.

Immunogenicity in vivo can be influenced by factors such as protein folding, post-translational modifications, and the immunogenetic background of host, which are not fully captured by current computational methods. Second, the tertiary structure and conformational dynamics of the multi-epitope construct in vivo may differ from the predicted models. For example, the flexibility and actual spatial arrangement of linkers, as well as the folding of the fused C-CPE domain, could affect epitope accessibility and processing by antigen-presenting cells. This may lead to differences in the exposure or immunodominance of certain epitopes compared to what is predicted in silico. Third, the possibility of unforeseen immunogenicity or allergenicity due to neo-epitopes formed at junctions or from the fusion of domains cannot be excluded. Additionally, the efficiency of mucosal delivery and the functional targeting of M cells by the C-CPE domain, while supported by computational docking, require empirical confirmation. Forth, cytokine induction analysis focused on IFN-γ and IL-4 as canonical markers of Th1/Th2 polarization; however, the exclusion of IL-17 and TNF-α—critical for T cell proliferation and cytotoxic responses—reflects a gap in predictive tool availability rather than biological relevance. Future work will experimentally profile these cytokines to address this limitation. Finally, computational tools rely on available databases and training data, which may not cover all HLA alleles or population diversity, potentially limiting the generalizability of our predictions. These factors underscore the necessity of in vitro and in vivo validation to refine construct efficacy.

## Conclusion

This study presents a novel multiepitope vaccine candidate with the potential to induce both systemic and mucosal immune responses against *B. pertussis*. By incorporating C-CPE as a mucosal delivery system and utilizing state-of-the-art immunoinformatics tools for epitope selection, we developed a vaccine that addresses the shortcomings of current acellular pertussis vaccines. Future research will focus on experimental validation and optimization to move this candidate toward clinical development. Despite these promising results, in vivo testing in animal models and clinical trials will be necessary to confirm its safety, efficacy, and ability to induce long-lasting immunity.

## Supplementary Information

Supplementary Material 1: Table S1. 730 CTL epitopes were identified from the S1 proteins.

Supplementary Material 2: Table S2. 12,744 CTL epitopes were identified from the F1 proteins.

Supplementary Material 3: Table S3. 6885 HTL epitopes (15-mers) were identified from the S1 proteins.

Supplementary Material 4: Table S4. 12,879 HTL epitopes (15-mers) were identified from the F1 proteins.

Supplementary Material 5: Table S5. 12 linear epitopes were identified from the S1 proteins.

Supplementary Material 6: Table S6. 16 linear epitopes were identified from the F1 proteins.

Supplementary Material 7: Table S7. The total numbers of initially predicted CTL, HTL, and B cell epitopes, as well as the numbers after filtration.

Supplementary Material 8: Table S8. NetChop predictions revealed several cleavage sites in the full construct.

Supplementary Material 9: Table S9. 498 HTL epitopes were identified from the C-CPE protein, based on reference sets of all HLA alleles.

Supplementary Material 10: Table S10 2472 CTL epitopes were identified from the C-CPE protein, based on reference sets of all HLA alleles.

## Data Availability

No datasets were generated or analysed during the current study.

## References

[1] Plotkin SA. The pertussis problem. Clin Infect Dis Off Publ Infect Dis Soc Am. 2014;58(6):830–3. 10.1093/cid/cit934 24363332

[2] Kapil P, Merkel TJ. Pertussis vaccines and protective immunity. Curr Opin Immunol. 2019;59:72–8. 31078081 10.1016/j.coi.2019.03.006 PMC6774807

[3] Dubois V, Locht C. Mucosal immunization against pertussis: lessons from the past and perspectives. Front Immunol. 2021;12:701285. 34211481 10.3389/fimmu.2021.701285 PMC8239240

[4] Solans L, Locht C. The role of mucosal immunity in pertussis. Front Immunol. 2018;9:3068. 30692990 10.3389/fimmu.2018.03068 PMC6339907

[5] Lee SF, Halperin SA, Salloum DF, MacMillan A, Morris A. Mucosal immunization with a genetically engineered pertussis toxin S1 fragment-cholera toxin subunit B chimeric protein. Infect Immun. 2003;71(4):2272–5. 12654855 10.1128/IAI.71.4.2272-2275.2003 PMC152103

[6] Couch RB. Nasal vaccination, Escherichia coli enterotoxin, and Bell’s palsy. N Engl J Med. 2004;350(9):860–1. 14985482 10.1056/NEJMp048006

[7] Neutra MR, Kozlowski PA. Mucosal vaccines: the promise and the challenge. Nat Rev Immunol. 2006;6(2):148–58. 16491139 10.1038/nri1777

[8] Li M, Wang Y, Sun Y, Cui H, Zhu SJ, Qiu HJ. Mucosal vaccines: strategies and challenges. Immunol Lett. 2020;217:116–25. 31669546 10.1016/j.imlet.2019.10.013

[9] Chasaide CN, Mills KHG. Next-generation pertussis vaccines based on the induction of protective T cells in the respiratory tract. Vaccines. 2020;8(4):621. 33096737 10.3390/vaccines8040621 PMC7711671

[10] Kim SH, Lee KY, Jang YS. Mucosal immune system and m cell-targeting strategies for oral mucosal vaccination. Immune Netw. 2012;12(5):165–75. 23213309 10.4110/in.2012.12.5.165 PMC3509160

[11] Suzuki H, Watari A, Hashimoto E, Yonemitsu M, Kiyono H, Yagi K, et al. C-terminal clostridium perfringens enterotoxin-mediated antigen delivery for nasal pneumococcal vaccine. PLoS ONE. 2015;10(5):e0126352. 26018248 10.1371/journal.pone.0126352 PMC4446347

[12] Kakutani H, Kondoh M, Fukasaka M, Suzuki H, Hamakubo T, Yagi K. Mucosal vaccination using claudin-4-targeting. Biomaterials. 2010;31(20):5463–71. 20398936 10.1016/j.biomaterials.2010.03.047

[13] Horvath D, Temperton N, Mayora-Neto M, Da Costa K, Cantoni D, Horlacher R, et al. Novel intranasal vaccine targeting SARS-CoV-2 receptor binding domain to mucosal microfold cells and adjuvanted with TLR3 agonist Riboxxim™ elicits strong antibody and T-cell responses in mice. Sci Rep. 2023;13(1):4648. 36944687 10.1038/s41598-023-31198-3 PMC10029786

[14] Lo DD, Ling J, Eckelhoefer AH. M cell targeting by a Claudin 4 targeting peptide can enhance mucosal IgA responses. BMC Biotechnol. 2012;13(12):7. 10.1186/1472-6750-12-7 PMC3337280 22413871

[15] Souod N, Rismani E, Bahrami F, Pakzad SR, Ajdary S. Computational evaluation of a fusion protein consisted of pertussis toxin and filamentous hemagglutinin from Bordetella pertussis to target Claudin-4 using C-terminal fragment of Clostridium perfringens enterotoxin. J Biomol Struct Dyn. 2021;39(16):5910–9. 32691700 10.1080/07391102.2020.1794966

[16] Feng Y, Jiang H, Qiu M, Liu L, Zou S, Li Y, et al. Multi-epitope vaccine design using an immunoinformatic approach for SARS-CoV-2. Pathog Basel Switz. 2021;10(6):737. 10.3390/pathogens10060737 PMC8230658 34208061

[17] Naz A, Shahid F, Butt TT, Awan FM, Ali A, Malik A. Designing multi-epitope vaccines to combat emerging coronavirus disease 2019 (COVID-19) by employing immuno-informatics approach. Front Immunol. 2020;11:1663. 32754160 10.3389/fimmu.2020.01663 PMC7365865

[18] SamimiHashjin A, Sardari S, Rostamian M, Ahmadi K, Madanchi H, Khalaj V. A new multi-epitope vaccine candidate based on S and M proteins is effective in inducing humoral and cellular immune responses against SARS-CoV-2 variants: an in silico design approach. J Biomol Struct Dyn. 2023;24:1–18. 10.1080/07391102.2023.2270699 37874075

[19] Doytchinova IA, Flower DR. VaxiJen: a server for prediction of protective antigens, tumour antigens and subunit vaccines. BMC Bioinformatics. 2007;5(8):4. 10.1186/1471-2105-8-4 PMC1780059 17207271

[20] Xue LC, Rodrigues JP, Kastritis PL, Bonvin AM, Vangone A. PRODIGY: a web server for predicting the binding affinity of protein-protein complexes. Bioinforma Oxf Engl. 2016;32(23):3676–8. 10.1093/bioinformatics/btw514 27503228

[21] Abraham MJ, Murtola T, Schulz R, Páll S, Smith JC, Hess B, et al. GROMACS: High performance molecular simulations through multi-level parallelism from laptops to supercomputers. SoftwareX. 2015;1(1–2):19–25.

[22] Torkashvand A, Bahrami F, Adib M, Ajdary S. Subcutaneous administration of a fusion protein composed of pertussis toxin and filamentous hemagglutinin from Bordetella pertussis induces mucosal and systemic immune responses. Iran J Basic Med Sci. 2018;21(7):753–9. 30140416 10.22038/IJBMS.2018.29112.7026 PMC6098962

[23] González-Mariscal L, Posadas Y, Miranda J, Uc PY, Ortega-Olvera JM, Hernández S. Strategies that target tight junctions for enhanced drug delivery. Curr Pharm Des. 2016;22(35):5313–46. 27510485 10.2174/1381612822666160720163656

[24] Zhang L. Multi-epitope vaccines: a promising strategy against tumors and viral infections. Cell Mol Immunol. 2018;15(2):182–4. 28890542 10.1038/cmi.2017.92 PMC5811687

[25] RoohparvarBasmenj E, Izadkhah H, Hosseinpour M, Saburi E, AbhajiEzabadi M, Alipourfard I. A novel approach to design a multiepitope peptide as a vaccine candidate for Bordetella pertussis. J Biomol Struct Dyn. 2023;8:1–13. 10.1080/07391102.2023.2278081 37937610

[26] Kar T, Narsaria U, Basak S, Deb D, Castiglione F, Mueller DM, et al. A candidate multi-epitope vaccine against SARS-CoV-2. Sci Rep. 2020;10(1):10895. 32616763 10.1038/s41598-020-67749-1 PMC7331818

[27] Ponomarenko J, Bui HH, Li W, Fusseder N, Bourne PE, Sette A, et al. ElliPro: a new structure-based tool for the prediction of antibody epitopes. BMC Bioinform. 2008;2(9):514. 10.1186/1471-2105-9-514 PMC2607291 19055730

[28] Behmard E, Soleymani B, Najafi A, Barzegari E. Immunoinformatic design of a COVID-19 subunit vaccine using entire structural immunogenic epitopes of SARS-CoV-2. Sci Rep. 2020;10(1):20864. 33257716 10.1038/s41598-020-77547-4 PMC7704662

[29] Arai R. Design of helical linkers for fusion proteins and protein-based nanostructures. Methods Enzymol. 2021;647:209–30. 33482989 10.1016/bs.mie.2020.10.003

[30] Bui HH, Sidney J, Li W, Fusseder N, Sette A. Development of an epitope conservancy analysis tool to facilitate the design of epitope-based diagnostics and vaccines. BMC Bioinformatics. 2007;26(8):361. 10.1186/1471-2105-8-361 PMC2233646 17897458

